# Consideration of Therapeutic Plasma Exchange in Association With Inflammatory Lesions in ANCA-Associated Glomerulonephritis: A Real-World Retrospective Study From a Single Center

**DOI:** 10.3389/fimmu.2021.645483

**Published:** 2021-06-17

**Authors:** Désirée Tampe, Philipp Ströbel, Peter Korsten, Samy Hakroush, Björn Tampe

**Affiliations:** ^1^ Department of Nephrology and Rheumatology, University Medical Center Göttingen, Göttingen, Germany; ^2^ Institute of Pathology, University Medical Center Göttingen, Göttingen, Germany

**Keywords:** autoimmune diseases, ANCA-associated glomerulonephritis, therapeutic plasma exchange, acute kidney injury, renal replacement therapy, intensive care treatment, inflammatory lesions

## Abstract

Anti-neutrophil cytoplasmic antibody (ANCA)-associated vasculitis (AAV) is a systemic vasculitis, most frequently presenting as microscopic polyangiitis (MPA) or granulomatosis with polyangiitis (GPA). Pathogenic ANCAs trigger a deleterious immune response resulting in pauci-immune necrotizing and crescentic glomerulonephritis (GN). Standard therapeutical regimens include aggressive immunosuppressive therapy. Since some patients require renal replacement therapy (RRT) despite intensive immunosuppressive therapy, additional therapeutic plasma exchange (PEX) to deplete pathogenic ANCAs has been recommended but its value has recently been questioned. Because therapeutic decision making is crucial in these critically ill patients, we here aimed to identify inflammatory lesions in association with PEX consideration in a retrospective study from a single center tertiary hospital in a real-world population of 46 patients with severe AAV requiring intensive care treatment. The decision to consider PEX was more likely in patients with need for intensive care treatment and severe renal dysfunction. In contrast, short-term outcomes did not depend on clinical, or laboratory characteristics assessed at admission. Histopathological analysis confirmed active disease reflected by increased glomerular necrosis and crescents, but these histopathological findings did not associate with short-term outcome either. Interestingly, only increased global glomerular sclerosis in renal biopsies associated with a detrimental short-term outcome. In conclusion, our study investigated determinants for the consideration of therapeutic PEX in patients with severe AAV requiring intensive care treatment. This aspect underscores the need for renal biopsy and requires further investigation in a prospective controlled setting for therapeutic decision making especially in patients with severe AAV requiring intensive care treatment, especially important for treating intensivists.

## Introduction

Anti-neutrophil cytoplasmic antibody (ANCA)-associated vasculitis (AAV) is a systemic vasculitis, which most frequently presents as microscopic polyangiitis (MPA) or granulomatosis with polyangiitis (GPA) (1). Renal involvement is a common and severe complication of AAV as it can cause requirement of renal replacement therapy (RRT), end-stage renal disease (ESRD) or death (2, 3). Pathogenic ANCAs trigger a deleterious immune response resulting in pauci-immune necrotizing and crescentic glomerulonephritis (GN), a typical manifestation of glomerular injury in AAV (4). Standard therapeutical regimens include aggressive immunosuppressive therapy to improve outcome in severe AAV (5). Since some patients require RRT despite intensive immunosuppressive therapy, additional therapeutic plasma exchange (PEX) to deplete pathogenic ANCAs has been recommended in patients with severe deterioration of kidney function due to rapidly progressive glomerulonephritis in new onset or relapse of disease, and for the treatment of severe diffuse alveolar hemorrhage (5–9). The MEPEX trial demonstrated that when compared with intravenous methylprednisolone, PEX increased the rate of renal recovery in AAV that presented with severe deterioration of kidney function (9). However, long-term outcomes (death or ESRD) did not differ among treatment groups (10). These observations were recently confirmed by the largest clinical trial of AAV to date (PEXIVAS), reporting no long-term benefit in outcomes (death or ESRD) of patients who received PEX in addition to standard immunosuppressive therapy (11). However, inclusion of patients with less severe renal dysfunction may limit the trial’s ability to apply these findings in a subgroup of critically ill patients with severe deterioration of kidney function at risk for requirement of RRT and death which we regularly see at our center. Indeed, PEXIVAS seems to confirm data from MEPEX that PEX, at least temporarily, can reduce the risk of ESRD, which is especially important for treating intensivists (11). Because therapeutic decision making is crucial in these critically ill patients, we here aimed to identify determinants for PEX consideration in a retrospective study from a single center tertiary hospital in a previously described real-world population of severe AAV requiring intensive care treatment.

## Methods

### Study Population

**Figure 1:**
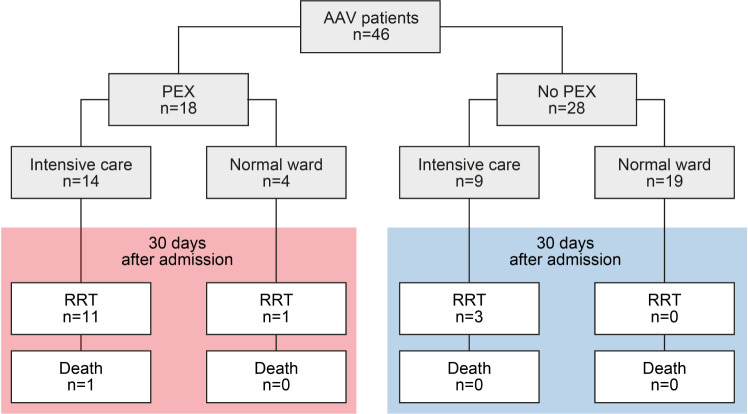
Total patient cohort of severe AAV. STROBE flow chart of patient disposition, RRT was performed intermittently in all cases. AAV, ANCA-associated vasculitis; STROBE, Strengthening the Reporting of Observational Studies in Epidemiology.

A total number of 46 patients with biopsy-proven AAV at the University Medical Center Göttingen were retrospectively included between 2015 and 2020, the patient cohort has, in part, previously been described (12–15). While no formal approval was required for the use of routine clinical data, a favorable ethical opinion was granted by the local Ethics committee (nos. 22/2/14 and 28/9/17). A detailed Strengthening the Reporting of Observational Studies in Epidemiology (STROBE) flow chart of patient disposition is shown in **Figure 1**, the patient cohort with severe AAV requiring intensive care treatment has previously been described (12). Medical records were used to obtain data on age, sex, diagnosis (MPO or PR3) and laboratory results (serum creatinine, C-reactive protein/CRP, urinary albumin/creatinine ratio). The estimated glomerular filtration rate (eGFR) was calculated using the Chronic Kidney Disease Epidemiology Collaboration (CKD-EPI) equation (16).

### Definitions

At admission, the Birmingham Vasculitis Activity Score (BVAS) version 3 was calculated as described previously (17). The BVAS is assessed on a scale of 0 to 63, with a score of 0 indicating the absence of disease activity and higher scores indicating active disease. The simplified acute physiology score (SAPS) II was calculated according to published guidelines (18). Requirement of intensive care treatment was defined by admission to the intensive care unit (ICU) or intermediate care unit (IMC) and calculated by the time from admission to relocation to a non-ICU/non-IMC medical ward. All patients required critical care treatment >24 h, RRT was performed intermittently in all cases. Indications for RRT included serum creatinine ≥500 μmol/L, severe electrolyte or acid-base abnormalities, volume overload or uremic encephalopathy. RRT was terminated when the eGFR surpassed 15 ml/min/1.73 m^2^ in the absence of hyperkalemia, heart failure, edema or uremic encephalopathy. Pulmonary hemorrhage was mild in all cases without requirement for mechanical ventilation.

### Renal Histopathology

Two renal pathologists (PS and SH) independently evaluated kidney biopsies and were blinded to data analysis. Within a renal biopsy specimen, each glomerulus was scored separately for the presence of necrosis, crescents and global sclerosis. Consequently, the percentage of glomeruli with any of these features was calculated as a fraction of the total number of glomeruli in each renal biopsy. Apart from these categories, the degree of interstitial fibrosis/tubular atrophy (IF/TA) was quantified. Based on these scorings, histopathological subgrouping according to Berden et al. (focal, crescentic, mixed or sclerotic class) and ARRS according to Brix et al. (low, medium or high risk) were performed (19, 20). Renal biopsies were also evaluated analogous to the Banff scoring system for allograft pathology (21).

### Remission Induction Therapy

PEX was administered during the induction period at the discretion of the treating physicians. Glucocorticoids (GCs) were administered either as intravenous pulse therapy or orally with a tapering schedule. Choice of further remission induction therapy was dependent on previous regimens and individual patients with preference for cyclophosphamide (CYC) in patients with severe deterioration of kidney function, a higher likelihood to choose rituximab (RTX) in younger patients with toxicity of CYC being the main reason for this choice (22). RTX was administered as four intravenous doses at 375 mg/m^2^ every week, RTX was not administered within 48 h before PEX treatment. As per our practice, PEX treatment was scheduled at least 48 h after RTX administration to avoid interference with the rapid immunosuppressive effects of RTX on circulating CD19-positive/CD20-positive lymphocytes as described previously (23, 24). CYC was administered as three intravenous doses up to 15 mg/kg every two weeks followed by one infusion every three weeks for three to six months, adjusted for age and renal function according to the CYCLOPS protocol (25). Combination therapy was administered as four intravenous doses at 375 mg/m^2^ RTX every week and two intravenous doses at 15 mg/kg CYC during the first and third RTX infusion. Prophylaxis to prevent pneumocystis (carinii) jiroveci infection was administered according to local practice.

### Statistical Methods

Variables were tested for normal distribution using the Shapiro–Wilk test. Non-normally distributed continuous variables are expressed as median and interquartile range (IQR), categorical variables are presented as frequency and percentage. Statistical comparisons were not formally powered or prespecified. For group comparisons, the Mann–Whitney U-test was used to determine differences in medians. Non-parametric between-group-comparisons were performed with Pearson’s Chi-square test. Data analyses were performed with GraphPad Prism (version 8.4.3 for MacOS, GraphPad Software, San Diego, California, USA).

## Results

**Figure 2:**
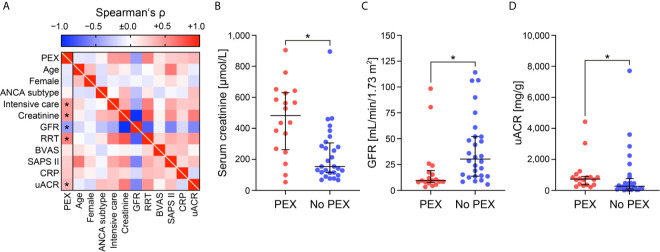
Clinical and laboratory determinants at admission to consider PEX for treatment of severe AAV. **(A)** Association between initiation of PEX treatment and clinical/laboratory findings are shown by heatmap reflecting mean values of Spearman’s ρ, asterisks indicate *p <0.05*. **(B–D)** The scatter dot plots represent medians and IQR with individual data points summarizing association between initiation of PEX treatment and indicated clinical/laboratory findings, Mann–Whitney U-test was used to determine differences in medians and asterisks indicate *p <0.05*. AAV, ANCA-associated vasculitis; ANCA, anti-neutrophil cytoplasmic antibodies; BVAS, Birmingham Vasculitis Activity Score; CRP, C-reactive protein; GFR, glomerular filtration rate (CKD-EPI); IQR, interquartile range; PEX, therapeutic plasma exchange; RRT, renal replacement therapy; SAPS II, simplified acute physiology score II; uACR, urinary albumin/creatinine ratio.

**Figure 3:**
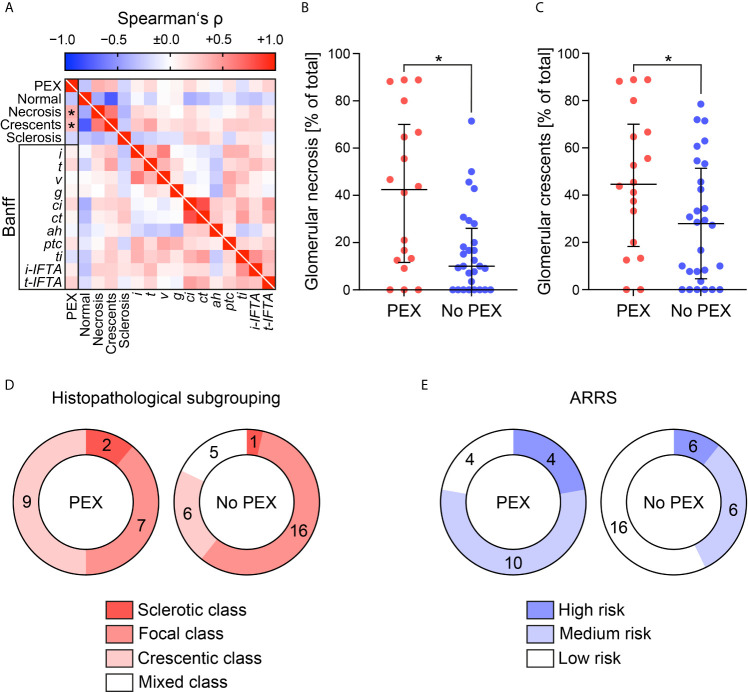
Renal histopathological findings in association with PEX for treatment of severe AAV. **(A)** Association between initiation of PEX treatment and renal histopathological findings are shown by heatmap reflecting mean values of Spearman’s ρ, asterisks indicate *p <0.05*. **(B, C)** The scatter dot plots represent medians and IQR with individual data points summarizing association between initiation of PEX treatment and indicated histopathological findings, Mann-Whitney U-test was used to determine differences in medians and asterisks indicate *p <0.05*. **(D, E)** Association between initiation of PEX treatment, histopathological subgrouping and ARRS are shown. AAV, ANCA-associated vasculitis; *ah*, arteriolar hyalinosis; ARRS, ANCA renal risk score; *ci*, interstitial fibrosis; *ct*, tubular atrophy; *g*, glomerulitis; GN, glomerulonephritis; *i*, interstitial inflammation; *i-IFTA*, inflammation in IFTA; IQR, interquartile range; PEX, therapeutic plasma exchange; *t*, tubulitis; *ptc*, peritubular capillaritis; *ti*, total inflammation; *t-IFTA*, tubulitis in IFTA; *v*, intimal arteritis.

**Figure 4:**
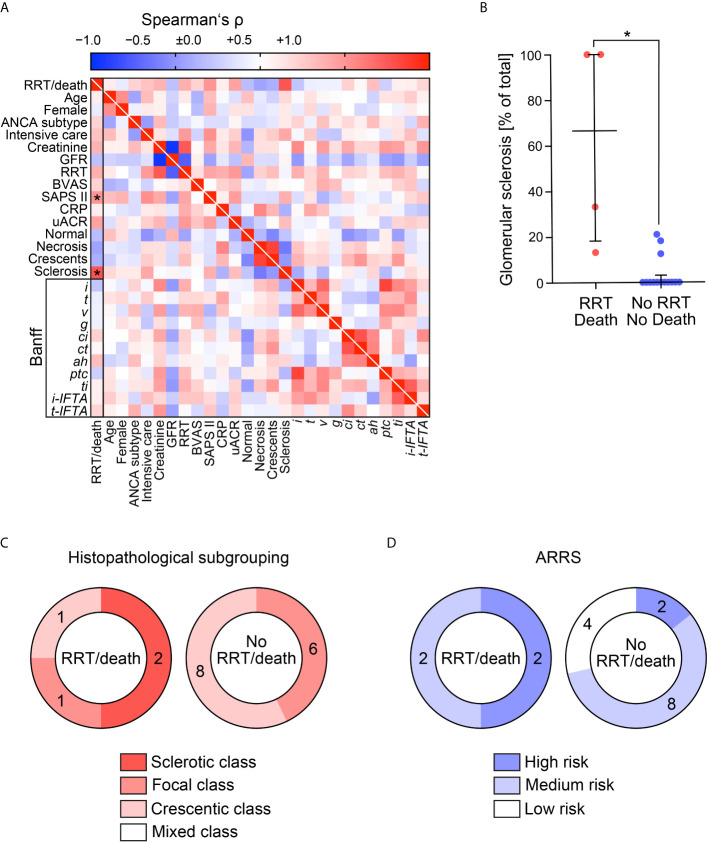
Determinants of short-term outcomes in patients with severe AAV receiving PEX treatment defined by the requirement of RRT at day 30 after admission or death within 30 days after admission. **(A)** Association with short-term outcome of patients with severe AAV receiving PEX treatment defined by requirement of RRT at day 30 after admission or death within 30 days after admission are shown by heatmap reflecting mean values of Spearman’s ρ, asterisks indicate *p <0.05*. **(B)** The scatter dot plots represent medians and IQR with individual data points summarizing association between short-term outcome of patients (RRT/death) and indicated histopathological findings, Mann–Whitney U-test was used to determine differences in medians and asterisks indicate *p <0.05*. **(C, D)** Association between short-term outcome of patients (RRT/death), histopathological subgrouping and ARRS are shown. AAV, ANCA-associated vasculitis; ANCA, anti-neutrophil cytoplasmic antibodies; BVAS, Birmingham Vasculitis Activity Score; CRP, C-reactive protein; GFR, glomerular filtration rate (CKD-EPI); IF/TA, interstitial fibrosis/tubular atrophy; IQR, interquartile range; PEX, therapeutic plasma exchange; RRT, renal replacement therapy; SAPS II, simplified acute physiology score II; uACR, urinary albumin/creatinine ratio.

At the discretion of the treating physicians, 18/46 (39.1%) patients with severe AAV received PEX (**Figure 1**). First, we analyzed clinical and laboratory characteristics at presentation for an association with the decision to initiate therapeutic PEX treatment. Our data revealed that the choice to consider PEX was more likely in patients with the need for intensive care treatment, severe renal dysfunction, requirement of RRT and elevated levels of urinary albumin/creatinine ratio (uACR, **Figures 2A–D** and **Table 1**). In contrast, immunosuppressive remission induction therapy and death within 30 days after admission did not differ between groups (PEX: one death due to sigmoid perforation, no PEX: two deaths due to septic shock) did not differ between groups (**Table 1**). Because severe renal dysfunction and elevated uACR indicates active and more aggressive kidney disease in AAV, we next correlated histopathological findings with former decision to initiate therapeutic PEX for treatment of severe AAV (26). Histopathological findings did not influence the decision to consider PEX for treatment of severe AAV since renal biopsy was performed after PEX initiation in most cases (**Table 2**). Among patients with severe renal dysfunction treated with PEX, histopathological analysis revealed active disease indicated by an increased fraction of glomeruli affected by necrosis and crescents compared to patients without therapeutic PEX treatment (**Figures 3A–C** and **Table 2**). This is also reflected by histopathological subgrouping and ARRS, patients with severe renal dysfunction treated with PEX were classified either into Berden’s focal/crescentic classes or ANCA renal risk score (ARRS) high/medium risk (**Figures 3D, E** and **Table 2**) (19, 20). In contrast, interstitial inflammatory lesions did not differ between both groups (**Figure 3A** and **Table 2**), confirming that severe renal dysfunction and PEX treatment is associated with glomerular manifestation of AAV. We next determined the short-term outcomes of patients with severe AAV receiving PEX treatment defined by the requirement of RRT at day 30 after admission or death within 30 days after admission. Both groups did not differ in clinical, or laboratory characteristics assessed at admission, limiting usability for association with short-term outcomes (**Figure 4A** and **Table 3**). Interestingly, an increased fraction of glomeruli with global glomerular sclerosis was associated with detrimental short-term outcomes (RRT/death, **Figures 4A, B** and **Table 3**). Berden’s histopathological subgrouping into sclerotic class along with ARRS high/medium risk classification identified patients for a detrimental short-term disease course (**Figures 4C, D** and **Table 3**), both considered to also show poorest long-term renal survival rates (19, 20). These findings indicate that although the decision to consider PEX in severe AAV was more likely in patients with severe renal dysfunction and active disease confirmed by histopathological analysis, there was no association of any clinical, laboratory or histopathological parameter for active disease with short-term outcomes. Although the choice for therapeutic PEX treatment in severe AAV did not associate with global glomerular sclerosis, this latter histopathological finding was associated with short-term outcomes.

**Table 1:** Characteristics of patients: PEX versus no PEX in severe AAV.

	*PEX (n = 18)*	*No PEX (n = 28)*	*P value*
Median sessions of PEX (IQR)—no.	5 (5–5.5)	*NA*	
*Clinical data*			
Median age (IQR)—years	62 (52.3–70)	69 (52–76)	*0.4506*
Female sex—no. (%)	6 (33.3)	13 (46.4)	*0.3787*
ANCA subtype MPO/PR3—no. (%)	10/8 (55.6/44.4)	14/14 (50/50)	*0.7128*
History of vasculitis—no. (%)	1 (2.2)	5 (10.9)	*0.2266*
Intensive care treatment—no. (%)	14 (77.8)	9 (32.1)	***0.0025***
Median intensive care treatment (IQR)—days	4 (1.5–8)	0 (0–2.75)	***0.0012***
Death within 30 days after admission—no. (%)	1 (5.6)	2 (7.1)	*0.8315*
*Renal injury*			
Median serum creatinine (IQR)—µmol/L	483 (263–631)	155 (114–306)	***0.0008***
Serum creatinine ≥500 µmol/L—no. (%)	9 (50)	1 (3.6)	***0.0002***
Median GFR (IQR)—ml/min/1.73 m^2^	9.6 (7.7–19.2)	30.4 (13.9–52.1)	***0.0011***
RRT—no. (%)	12 (66.7)	3 (10.7)	***<0.0001***
Median RRT (IQR)—days	4 (0–11.8)	0 (0–0)	***0.0002***
*Extrarenal manifestations*			
Pulmonary hemorrhage—no. (%)	3 (16.7)	3 (10.7)	*0.5585*
Skin involvement—no. (%)	3 (16.7)	5 (17.9)	*0.9172*
*Disease activity*			
Median BVAS (IQR)—points	18 (16–20.5)	18 (15–20.8)	*0.4482*
Median SAPS II at admission (IQR)—points	26 (24–33)	22 (19–31.8)	*0.1086*
Median CRP (IQR)—mg/L	71.4 (42.4–178)	35.2 (10.7–100)	*0.0662*
Median uACR (IQR)—mg/g	728 (362-904)	273 (99.3–760)	***0.0187***
*Remission induction therapy*			
Intravenous steroid pulse—no. (%)	14 (77.8)	21 (75)	
Oral GCs—no. (%)	18 (100)	28 (100)	
RTX—no. (%)	5 (27.8)	11 (39.3)	
CYC—no. (%)	9 (50)	15 (53.6)	
RTX/CYC—no. (%)	4 (22.2)	2 (7.1)	
*Follow-up*			
Median follow-up (IQR)—days	468 (215–798)	221 (66–445)	*0.0697*
RRT—no. (%)	3 (16.7)	2 (7.1)	*0.3112*
Death—no. (%)	0 (0)	0 (0)	

For group comparisons, the Mann–Whitney U-test was used to determine differences between medians. Non-parametric between-group-comparisons were performed with Pearson’s Chi-square test. Bold indicates statistically significant values at group level. ANCA, anti-neutrophil cytoplasmic antibodies; BVAS, Birmingham Vasculitis Activity Score; CRP, C-reactive protein; GFR, glomerular filtration rate (CKD-EPI); IF/TA, interstitial fibrosis/tubular atrophy; IQR, interquartile range; MPO, myeloperoxidase; NA, not applicable; No., number; PEX, therapeutic plasma exchange; PR3, proteinase 3; RRT, renal replacement therapy; RTX, rituximab; SAPS II, simplified acute physiology score II; uACR, urinary albumin/creatinine ratio.

**Table 2:** Renal histopathological findings: PEX versus no PEX in severe AAV.

	*PEX (n = 18)*	*No PEX (n = 28)*	*P value*
Initiation of PEX before kidney biopsy—no. (%)	13 (72.2)	*NA*	
Initiation of PEX before kidney biopsy—days (IQR)	5 (0.5–9.5)	*NA*	
*Renal histology*			
Median total glomeruli (IQR)—no.	13 (8.75–17.3)	17 (11.3–31)	*0.0586*
Median normal glomeruli (IQR)—%	34.3 (11.6–2.3)	55.4 (36.5–7.6)	*0.0558*
Median glomerular necrosis (IQR)—%	42.5 (11.6–70)	10 (0–26)	***0.0119***
Median glomerular crescents (IQR)—%	44.6 (18.3–70)	27.9 (4.6–51.4)	***0.0364***
Median glomerular sclerosis (IQR)—%	0 (0–18.9)	9.2 (0–29.9)	*0.1755*
*Banff lesion*			
Interstitial inflammation: *i* (IQR)—score	0 (0–0.25)	0 (0–0)	*0.7010*
Tubulitis: *t* (IQR)—score	1 (0–1)	0 (0–1)	*0.2375*
Arteritis: *v* (IQR)—score	0 (0–1)	0 (0–1)	*0.9999*
Glomerulitis: *g* (IQR)—score	2 (0–2)	2 (1–2)	*0.7221*
Interstitial fibrosis: *ci* (IQR)—score	2 (1–2.25)	1 (1–2)	*0.2470*
Tubular atrophy: *ct* (IQR)—score	1 (1–2)	1 (1–2)	*0.7645*
Arteriolar hyalinosis *ah* (IQR)—score	0 (0–1.75)	0 (0–1)	*0.7510*
Peritubular capillaritis: *ptc* (IQR)—score	0 (0–0)	0 (0–0)	*0.6344*
Total inflammation: *ti* (IQR)—score	1 (0–1)	1 (0–1)	*0.3317*
Inflammation in IFTA: *i-IFTA* (IQR)—score	2 (1–3)	2 (1–3)	*0.5706*
Tubulitis in IFTA: *t-IFTA* (IQR)—score	1 (0.75–1)	1 (0–1)	*0.2252*
*Histopathological subgrouping*			
Sclerotic class—no. (%)	2 (11,1)	1 (3.6)	
Focal class—no. (%)	7 (38.9)	16 (57.1)	
Crescentic class—no. (%)	9 (50)	6 (21.4)	
Mixed class—no. (%)	0 (0)	5 (17.9)	*0.0540*
*ARRS*			
High risk—no. (%)	4 (22.2)	3 (10.7)	
Medium risk—no. (%)	10 (55.6)	9 (32.1)	
Low risk—no. (%)	4 (22.2)	16 (57.1)	*0.0646*

For group comparisons, the Mann–Whitney U-test was used to determine differences between medians. Non-parametric between-group-comparisons were performed with Pearson’s Chi-square test. Bold indicates statistically significant values at group level. AAV, ANCA-associated vasculitis; ARRS, ANCA renal risk score; IF/TA, interstitial fibrosis/tubular atrophy; IQR, interquartile range; No., number; PEX, therapeutic plasma exchange.

**Table 3:** Characteristics of patients receiving PEX: short-term outcome within 30 days.

	*RRT/death (n = 4)*	*No RRT/death (n = 14)*	*P value*
RRT at day 30 after admission—no. (%)	3 (75)	*NA*	
Death within 30 days after admission—no. (%)	1 (25)	*NA*	
*Clinical data*			
Median age (IQR)—years	64 (51.3–74.5)	62 (52.3–69.3)	*0.7389*
Female sex—no. (%)	1 (25)	5 (35.7)	*0.6885*
ANCA subtype MPO/PR3—no. (%)	3/1 (75/25)	7/7 (50/50)	*0.3749*
History of vasculitis—no. (%)	0 (0)	1 (7.1)	*0.5823*
Intensive care treatment—no. (%)	4 (100)	10 (71.4)	*0.2254*
Median intensive care treatment (IQR)—days	14.5 (25.5)	4 (0–6.5)	*0.0905*
*Renal injury*			
Median serum creatinine (IQR)—µmol/L	586 (460–893)	411 (228–596)	*0.1575*
Serum creatinine ≥500 µmol/liter—no. (%)	3 (75)	6 (42.9)	*0.2568*
Median GFR (IQR)—ml/min/1.73 m^2^	8.8 (4.6–10.3)	10.7 (7.9–24.6)	*0.2327*
*Disease activity*			
Median BVAS (IQR)—points	21 (15.8–24.8)	18 (16–19.3)	*0.4294*
Median SAPS II at admission (IQR)—points	37 (27–54.5)	24.5 (23.3–31.3)	***0.0422***
Median CRP (IQR)—mg/L	111 (33.3–204)	71.4 (42.4–117)	*0.7980*
Median uACR (IQR)—mg/g	1,940 (732–4,083)	685 (348–847)	*0.0791*
*Renal histology*			
Median total glomeruli (IQR)—no.	13 (8.75–15)	13.5 (8.75–18.3)	*0.6297*
Median normal glomeruli (IQR)—%	10 (0–60)	39.9 (17.9–62.3)	*0.2003*
Median glomerular necrosis (IQR)—%	6.7 (0–38.3)	49.7 (15.6–82.1)	*0.0755*
Median glomerular crescents (IQR)—%	23.3 (12.7–42.4)	54.1 (33.1–82.1)	*0.1170*
Median glomerular sclerosis (IQR)—%	66.7 (18.3–100)	0 (0–3.1)	***0.0013***
Median IF/TA (IQR)—%	40 (22.5–65)	20 (8.75–33.8)	*0.0925*
*Histopathological subgrouping*			
Sclerotic class—no. (%)	2 (50)	0 (0)	***0.0193***
Focal class—no. (%)	1 (25)	6 (42.9)	
Crescentic class—no. (%)	1 (25)	8 (57.1)	
*ARRS*			
High risk—no. (%)	2 (50)	2 (14.3)	*0.2280*
Medium risk—no. (%)	2 (50)	8 (57.1)	
Low risk—no. (%)	0 (0)	4 (28.6)	
*Follow-up*			
Median follow-up (IQR)—days	378 (97.3–	531 (215–798)	*0.5739*
RRT—no. (%)	1,164)	0 (0)	***0.0004***
Death – no. (%)	3 (75)	0 (0)	
	0 (0)		

For group comparisons, the Mann–Whitney U-test was used to determine differences between medians. Non-parametric between-group-comparisons were performed with Pearson’s Chi-square test. Bold indicates statistically significant values at group level. ANCA, anti-neutrophil cytoplasmic antibodies; BVAS, Birmingham Vasculitis Activity Score; CRP, C-reactive protein; GFR, glomerular filtration rate (CKD-EPI); IF/TA, interstitial fibrosis/tubular atrophy; IQR, interquartile range; MPO, myeloperoxidase; NA, not applicable; No., number; PEX, therapeutic plasma exchange; PR3, proteinase 3; RRT, renal replacement therapy; RTX, rituximab; SAPS II, simplified acute physiology score II; uACR, urinary albumin/creatinine ratio.

## Discussion

According to current recommendations, therapeutic PEX should be considered for AAV patients with severe deterioration of kidney function (serum creatine levels >500 µmol/L due to rapid-progressive GN in new onset or relapse of disease) and for the treatment of severe diffuse alveolar hemorrhage (6). It has been assumed that severe deterioration of renal function is associated with extensive amounts of chronic lesions for which PEX treatment would not likely be successful. However, recent studies revealed that the majority of patients presenting with severe kidney dysfunction have extensive acute lesions and that a significant number of patients benefit from PEX treatment (27). Most biopsies in patients with severe deterioration of kidney function had extensive acute lesions with crescents and necrosis, whereas the amount of global glomerular sclerosis was relatively low. Recently, the largest clinical trial of AAV to date (PEXIVAS) indicated no long-term improvement in renal outcomes (death or ESRD) of patients who received PEX in addition to standard immunosuppressive therapy (11). However, it is possible that treating intensivists were reluctant to enroll the most critically ill patients because of random assignment to the no PEX regimen, thus creating a selection bias. Therefore, inclusion of patients with less severe renal dysfunction may limit the trial’s ability to apply these findings to a subgroup of critically ill patients with severe deterioration of kidney function which we regularly see at our center (28). The largest trial performed in AAV with severe deterioration of kidney function (MEPEX) recruited patients with either a serum creatine >500 µmol/L or requirement of RRT (9). Therapeutic PEX appeared to prevent ESRD or death at 3 months, but long-term follow-up data revealed no statistically significant benefit for PEX treatment (9, 10). However, short-term outcomes are especially important for intensivists because a subgroup of patients with severe AAV need intensive care treatment at risk for requirement of RRT and death. Here, we provide the first study to address choice for therapeutic PEX in a real-world population of patients with severe AAV requiring intensive care treatment, especially important for treating intensivists. We here identified inflammatory lesions associated with severe renal dysfunction and PEX treatment in a retrospective study from a single center tertiary hospital of previously described patients with severe AAV requiring intensive care treatment (12). The decision to consider PEX was more likely in patients with need for intensive care treatment and severe renal dysfunction. In contrast, short-term outcome did not associate with clinical or laboratory characteristics assessed at admission. Histopathological analysis confirmed active disease reflected by increased glomerular necrosis and crescents, also reflected by histopathological subgrouping and ARRS (19, 20). In contrast, interstitial inflammatory lesions did not differ between both groups, confirming that severe renal dysfunction and PEX treatment is associated with glomerular manifestation of AAV. This is in line with our previous observation that the short-term renal outcome was associated with active glomerular lesions reflected by a lower fraction of normal glomeruli, increased glomerular necrosis and crescents (12). Conversely, only an increased fraction of glomeruli affected by global glomerular sclerosis in renal biopsies associated with a detrimental short-term outcome in patients with severe AAV receiving PEX treatment. Again, this is in line with our previous observation that presence of global glomerular sclerosis showed the strongest inverse relationship with short-term renal outcome in critically ill patients with AAV requiring intensive care treatment (12). Furthermore, these observations are in line with previous reports that chronic lesions, in both the glomerular and the interstitial compartments, are inversely correlated with renal recovery in AAV (29). Particularly, glomerular sclerosis and arteriosclerosis correlated with dialysis-dependent ESRD at one year follow-up in patients requiring RRT at diagnosis (29). It has been assumed that severe renal dysfunction is associated with excessive scarring and that aggressive immunosuppressive therapy including PEX cannot be successful in all AAV cases (8). This could be on part explained by the irreversibility of chronic lesions despite aggressive immunosuppressive therapy. However, AAV cases with low numbers of normal glomeruli and treated with therapeutic PEX may still recover from RRT (29). Our findings that renal biopsy findings enable to confirm both, active disease and scarring, further support early conduction of a renal biopsy whenever possible (28–32).

The main limitations of our study are its retrospective design, different regimens of remission induction, the small patient number, influence of therapeutic PEX on histopathological findings because PEX treatment was initiated before renal biopsy in most cases, and limited data on long-term renal survival rates. Nevertheless, the number of critically ill patients with AAV at our center is considerable and our study identified determinants for the consideration of therapeutic PEX in patients with severe AAV requiring intensive care treatment despite the negative results of the PEXIVAS trial. It has to be kept in mind, however, that the patients included in the PEXIVAS trial differed from the real-world population regularly admitted to our ICU. This new aspect underscores the need for renal biopsy and requires further investigation in a prospective controlled setting for therapeutic decision making and short-term care especially in patients with severe AAV requiring intensive care treatment, especially important for treating intensivists (28, 30–32).

## Conclusions

Renal involvement is a common and severe complication of AAV as it can cause requirement of RRT, ESRD or death. This study identifies determinants for PEX consideration in patients with severe AAV requiring intensive care treatment. This aspect underscores the need for renal biopsy especially important in patients with severe AAV requiring intensive care treatment.

## Data Availability Statement

The original contributions presented in the study are included in the article. Further inquiries can be directed to the corresponding author.

## Ethics Statement

The studies involving human participants were reviewed and approved by the Institutional Review Board of the University Medical Center Göttingen, Germany (no. 22/2/14 and 28/9/17). The patients/participants provided their written informed consent to participate in this study.

## Author Contributions

SH and BT conceived the study, collected and analyzed data and co-wrote the first draft. DT collected and analyzed data. PS and SH evaluated histopathological findings. PK analyzed data and edited the manuscript. SH and BT contributed equally as senior authors. All authors contributed to the article and approved the submitted version.

## Funding

BT was supported by the Research program, University Medical Center, University of Göttingen (1403720). The funding sources had no involvement in the design, collection, analysis, interpretation, writing or decision to submit the article.

## Conflict of Interest

The authors declare that the research was conducted in the absence of any commercial or financial relationships that could be construed as a potential conflict of interest.
